# Probability judgments under ambiguity and conflict

**DOI:** 10.3389/fpsyg.2015.00674

**Published:** 2015-05-20

**Authors:** Michael Smithson

**Affiliations:** Research School of Psychology, The Australian National UniversityCanberra, ACT, Australia

**Keywords:** judgment, probability, ambiguity, conflict, uncertainty

## Abstract

Whether conflict and ambiguity are distinct kinds of uncertainty remains an open question, as does their joint impact on judgments of overall uncertainty. This paper reviews recent advances in our understanding of human judgment and decision making when both ambiguity and conflict are present, and presents two types of testable models of judgments under conflict and ambiguity. The first type concerns estimate-pooling to arrive at “best” probability estimates. The second type is models of subjective assessments of conflict and ambiguity. These models are developed for dealing with both described and experienced information. A framework for testing these models in the described-information setting is presented, including a reanalysis of a multi-nation data-set to test best-estimate models, and a study of participants' assessments of conflict, ambiguity, and overall uncertainty reported by Smithson (2013). A framework for research in the experienced-information setting is then developed, that differs substantially from extant paradigms in the literature. This framework yields new models of “best” estimates and perceived conflict. The paper concludes with specific suggestions for future research on judgment and decision making under conflict and ambiguity.

## Introduction

Whether conflict and ambiguity are distinct kinds of uncertainty remains an open question, as does their joint impact on judgments of overall uncertainty. Several experimental judgment studies (Smithson, 1999, 2013; Cabantous, 2007; Cabantous et al., 2011; Baillon et al., 2012) support the claim that conflict and ambiguity are distinct. However, in some generalized probability frameworks that deal in sets of probabilities, this distinction appears unnecessary or irrelevant (an accessible survey of such frameworks is provided in Augustin et al., 2014). This paper reviews recent advances in our understanding of human judgment and decision making when both ambiguity and conflict are present, and presents new models and methodological suggestions regarding research on this topic.

There is some confusion regarding the concepts of ambiguity and conflict. In this paper, we take *ambiguity* to mean either a set of possible qualitative states or a range of possible values on a continuum. An example of the former is the meaning of “hot” in the statement “This food is hot,” where “hot” could mean “high temperature,” “spicy,” “stolen,” “sexy,” and so on. An example of the latter is an interval estimate, such as “My weight is somewhere between 74 and 75 kg.” We define *conflict* as referring to disagreements among sources about some aspect of reality. An example is one bushfire expert estimating the probability of a major bushfire in a particular woodland as “more than 0.5” while another bushfire expert estimates this probability as “less than 0.5.” Thus, ambiguity is a type of uncertainty that can arise from one message, whereas conflict is a type of uncertainty arising from multiple messages.

There are two types of settings for judgments and decisions under uncertainty, and the literature comparing them has almost exclusively dealt with judgments of probability. The first, and most widely studied, is the *described-information* setting. The judges or decision makers are presented with a description of the uncertainties inherent in their task (typically the probabilities of relevant events or outcomes). The second setting is the *experienced-information* setting (Rakow and Newell, 2010), wherein judges sample outcomes from an environment and build up their own assessments of event or outcome probabilities on the basis of their samples. There is debate concerning whether the heuristics people use for judging probabilities and making decisions in these two settings differ or are essentially the same. In this paper we will extend this debate to uncertainties arising from ambiguous and conflicting information.

Recently formal, quasi-rational, models of judgments under conflict and ambiguity have been proposed (Gajdos and Vergnaud, 2013; Smithson, 2013) with parameters to represent judges' orientations toward conflictive and ambiguous uncertainties. These models are amenable to empirical tests via their predictions of what human judges will do, and they can be applied to both described- and experienced-information settings, although they were developed in the first setting.

This paper presents a program for researching judgments when information is both conflicting and ambiguous. The section following this one discusses two types of testable models of judgments under conflict and ambiguity. The first type concerns estimate-pooling to arrive at “best” probability estimates. The second type is models of subjective assessments of conflict and ambiguity. These models are developed for dealing with both described and experienced information. The next section presents a framework in the described-information setting. It includes a reanalysis of a data-set to test best-estimate models, and a study of participants' assessments of conflict, ambiguity, and overall uncertainty reported by Smithson (2013). The section thereafter develops a framework for research in the experienced-information setting. The paper then concludes with a discussion of specific future directions for this line of research.

## Models

### “Best” estimates and preferences

We assume that *K* diagnostic judges provide estimates of a probability, of the form [*p*_*k*1_, *p*_*k*2_, …, *p*_*kJ*_], where the *p*_*kj*_ are order statistics: *p*_*k*1_ <*p*_*k*2_ <… <*p*_*kJ*_. The simplest setup of this kind, which we shall consider, has two judges, each of whom provides a lower and upper estimate, so that *K* = 2 and *J* = 2.

In some situations, decision makers (DMs) may wish to determine what the “best” estimate of this probability is, and/or their assessments regarding optimism or pessimism of the judges' estimates. For example, what is the “best” estimate of a probability, given the interval estimate [0.3, 0.6]? And if offered a second interval estimate of the same probability, say [0.2, 0.7], does one interval indicate a higher probability than the other and, if so, which one?

A conventional approach to described-information estimates of this kind is to assume a uniform distribution over the interval, so its midpoint is the “best” estimate and, in our example, both intervals yield identical best estimates. More generally, this “midpoint model” is a special case of the widely-used weighted arithmetic averaging method (or linear pool) for combining alternative estimates to arrive at a best estimate. According to this approach, if the event under consideration is a reward, then any DM who regards a value below the midpoint as the best estimate is pessimistic (or risk-averse) and a value above the midpoint indicates optimism (or risk-seeking).

A factor that, to my knowledge, has not been systematically considered in research on ambiguity, is the assumptions that the DM makes about how the interval has been constructed. Different assumptions can yield quite different pooling methods and results for the “best” estimate and, therefore, for inferences about DM risk orientations and comparisons among interval estimates. I will demonstrate this point with imprecise probability models from Walley (1991, p. 93–96).

Suppose the DM assumes that the interval reflects a tax on gambles of the kind that is used in some betting agencies. If a bookmaker regards *P*_*v*_ as the probability of a gamble paying off, then s/he will only offer to bet at a reduced rate, (1 – *w*) *P*_*v*_, where 0 < *w* < 1 and *w* is the profit-margin desired by the bookmaker. Thus, *P*_*v*_ is a coherent probability for which *p*_1_ and *p*_2_ are the corresponding lower and upper probabilities of the linear-vacuous mixture described by Walley. Setting *p*_2_ −*p*_1_ = *w*, we have *p*_1_ = (1 − *w*)*P*_*v*_ and *p*_2_ = (1 − *w*)*P*_*v*_ + *w*. So, when presented with the interval [*p*_1_, *p*_2_], our DM derives *P*_*v*_ as follows:

(1) $$P_{v}=\frac{p_{1}}{1-p_{2}+p_{1}}.$$

Denoting the interval midpoint by *P*_*m*_, it is easy to show that *P*_*v*_ = *P*_*m*_ iff *p*_1_ + *p*_2_ = 1, and otherwise *P*_*v*_ > (<)*P*_*m*_ ⇔ *p*_1_ + *p*_2_ > (<)1. For our example intervals, [0.3, 0.6] and [0.2, 0.7], *P*_*m*_ = 0.45 in both cases, whereas for [0.3, 0.6] *P*_*v*_ = 0.429 and for [0.2, 0.7] *P*_*v*_ = 0.4. A midpoint DM arrives at identical “best estimates” both intervals, whereas the vacuous-linear DM obtains a higher “best estimate” for [0.3, 0.6] than for [0.2, 0.7]. The contrast between the midpoint and linear-vacuous models is greatest when either interval limit approaches its bound on the unit interval. From Equation (1) it is clear that as *p*_1_ approaches 0, *P*_*v*_ also approaches 0; and *P*_*v*_ approaches 1 as *p*_2_ approaches 1.

If two intervals with identical midpoints do not generally produce identical *P*_*v*_-values, what pair of intervals will do so? Given two intervals, [*p*_1_, *p*_2_] and [*q*_1_, *q*_2_], a bit of algebra suffices to show that their *P*_*v*_-values are identical when *p*_1_/(1 − *p*_2_). = *q*_1_/(1 − *q*_2_). Thus, in contrast with our previous example of the midpoint-equivalent pair of intervals [0.3, 0.6] and [0.2, 0.7], the vacuous-linear DM's pair would be [0.3, 0.6] and [0.15, 0.8].

A different taxation scheme provides different best estimates. Another example from Walley (1991, p. 95–96) is a “capital gains tax” setup, in which *p*_1_ = (1 − *t*)*P*_*c*_/ (1 − *tP_c_*) and *p*_2_ = *P*_*c*_/(1 − *tP_c_*), where *P*_*c*_ is the best estimate for this model and 0 < *t* < 1. In this scheme, we have

(2) $$\begin{array}{c} P_{c}=\frac{p_{2}}{1+p_{2}-p_{1}}\\ t=\frac{p_{2}-p_{1}}{p_{2}} \end{array}$$

It is easy to show that *P*_*c*_ = *P*_*v*_ iff *p*_1_ + *p*_2_ = 1; otherwise *P*_*c*_ > (<) *P_v_* ⇔ *p*_1_ + *p*_2_ < (>) 1, and that *P*_*m*_ falls in between them.

Indefinitely many taxation schemes can be invented (e.g., the Parimutuel system that is popular in horse-racing, or changing the scale of what is being taxed, as in a linear-vacuous model that taxes the probability of winning in the logit scale). There are two main points to this material. First, the rational “best” estimate for a probability interval depends on how the DM believes interval has been constructed. We shall see this point playing a crucial part in the development of a framework for research on ambiguity and conflict from experience. Second, the fact that there are multiple models for best estimates enriches the study of how humans make their own best estimates, both by providing a variety of models to compare with human performance and by cautioning researchers against simplistic imputations of “risk-aversion” or “risk-seeking” to estimates that deviate from the interval midpoint. It also is possible that people may use methods for pooling conflicting estimates that differ from those they use for pooling ambiguous ones.

### Models of assessments of ambiguity and conflict

Given the setup discussed in the preceding subsection, the *k*^th^ judge's assessment is ambiguous insofar as the *p*_*kj*_ diverge in some sense from one another, and we will consider functions *A*(*p*_*kj*_) to measure ambiguity. Likewise, judges' assessments may conflict with one another insofar as their assessments differ in some sense from each other, and we will also consider functions *C*(*p*_*kj*_) to measure conflict. Finally, a decision maker (DM) who is given these judges' assessments may have a subjective appraisal of the combined uncertainty resulting from both ambiguity and conflict that weighs these two uncertainty components according to their relative aversiveness for the DM. We will therefore investigate uncertainty functions *S*(α, θ, *C*(*p*_*kj*_), *A*(*p*_*kj*_)) that are monotonically increasing in *C*(*p*_*kj*_) and *A*(*p*_*kj*_), where α is the conflict weight and θ is the ambiguity weight.

Both variance and distance are reasonable uncertainty metrics for both ambiguity and conflict. Ambiguity effects on judgments and decisions have been explained in terms of variance (Rode et al., 1999), and conflict also has implications for variability in outcomes. For the models described here, the variance and distance versions are equivalent, so we shall restrict attention to the variance version. The ambiguity of each judge's estimates can be measured by

(3) $$A_{k}=\sum_{j=1}^{J}{\left(p_{kj}-\bar{p_{k.}}\right)}^{2}/J,$$

so that the total ambiguity is just the within-judge component of the variance of the *p*_*kj*_:

$$A=\sum_{k=1}^{K}A_{k}/K,$$

where *p*_*k*._ is the mean judgment for the *k*^th^ judge. The between-judge variance component is an intuitively plausible candidate for measuring conflict:

(4) $$C_{1}=\sum_{k=1}^{K}{\left(\bar{p_{k.}}-\bar{p_{..}}\right)}^{2}/K,$$

where *p*_.._ is the grand mean. However, an alternative conflict measure is the variance among the order-statistics of the same rank (i.e., the variance of the *p*_*kj*_ around *p*_*.j*_):

(5) $$C_{2}=\sum_{k=1}^{K}\sum_{j=1}^{J}{\left(p_{kj}-\bar{p_{.j}}\right)}^{2}/JK.$$

I shall refer to the first model (Equation 4) as variance component model 1 (VC1) and the second (Equation 5) as VC2. The conflict function in Equation (5) differs from that in Equation (4) in an important way, because when *p*_*k*._ are identical for all *K* judges, *C*_1_ = 0 whereas this is not true for *C*_2_. Thus, VC1 predicts that a pair of interval estimates with identical midpoints will not be perceived by the DM as conflictive, whereas VC2 predicts that they will be.

Gajdos and Vergnaud (2013) present a model of decision making under ambiguity and conflict based on the Gilboa and Schmeidler (1989) maxmin framework. They intended their model to apply to probability judgments; Smithson (2013) extends it to judgments of magnitudes and describes the two-state, two-judge special case of their model. This version of the Gajdos–Vergnaud (GV) model is reproduced here.

In the GV model, the α and θ weights are used to modify the order statistics of each judge. The θ parameter contracts the [*p*_*k*1_, *p*_*k*2_] interval around its midpoint at a rate 1−θ, yielding lower and upper bounds.

(6) $$\begin{array}{c} \pi_{k1}=p_{k1}(1+\theta )/2+p_{k2}(1-\theta )/2,\\ \pi_{k2}=p_{k1}(1-\theta )/2+p_{k2}(1+\theta )/2. \end{array}$$

Gajdos and Vergnaud do not define an ambiguity measure along the lines of those in this paper, but as with the variance and distance models we may construct one by summing the differences π_*k*2_ −π_*k*1_. Smithson (2013) shows that this measure is a simple function of the ambiguity measure in a distance model.

The GV model treats α as contracting the pairs of interval endpoints *p*_*kj*_ and *p*_*mj*_ (i.e., the *k*^th^ and *m*^th^ judges' estimates of the *j*^th^ endpoint) around their mean at the rate1−α. Thus, the order statistics are modified in the following way:

(7) $$\begin{array}{c} \gamma_{kj}=p_{kj}(1+\alpha )/2+p_{mj}(1-\alpha )/2,\\ \gamma_{mj}=p_{mj}(1+\alpha )/2+p_{kj}(1-\alpha )/2. \end{array}$$

Again, Gajdos and Vergnaud do not define a conflict measure but one may be defined by summing the absolute values of the differences γ_*kj*_ −γ_*mj*_. Smithson (2013) shows that this measure is a simple function of a *C*_2_ measure in a distance model.

The models of ambiguity and conflict developed here are testable, and they differ in their predictions of how people will assess ambiguity, conflict, and thus overall uncertainty when they are presented with alternative probability estimates of the same event. They are amenable to being tested in both described- and experienced-information settings.

## Ambiguity and conflict in described-information settings

Ambiguity has been widely studied in experimental judgment and decision making research, beginning with Ellsberg's (1961) classic experiments. The most common description of an ambiguous probability in these experiments is an interval (e.g., “the probability of event *E* is somewhere between 0.2 and 0.5,” or “the probability of event *E* is unknown”). To my knowledge, no studies have involved disjoint sets of probabilities (e.g., “the probability of event *E* is either 0.2 or 0.5”). Indeed, the distinction between alternative discrete possible states and a continuous range of values for some quantity is largely absent from the psychological literature (but see Guney and Newell, 2014).

Conflict, on the other hand, readily yields disjoint sets of probabilities (e.g., “expert A estimates the probability of event *E* to be 0.2 whereas expert B estimates it to be 0.5”). Several studies have compared people's assessments of uncertainty arising from ambiguity vs. conflict, with a general result that people seem to prefer ambiguous but agreeing risk messages to unambiguous but conflicting messages (Smithson, 1999; Cabantous, 2007; Baillon et al., 2012). However, there have been very few attempts to systematize such comparisons.

### Study 1: best estimates

To date, there are no studies of how people pool estimates that are both ambiguous and conflicting. However, we may put the three best-estimate models developed in the preceding sections (the midpoint, linear-vacuous, and capital-gains models) to an empirical test in a described-information setting where ambiguity is present. The dataset for this purpose is the product of a 25-sample, 24-nation, 17-language study of laypeople's interpretations of the probability expressions that the Intergovernmental Panel on Climate Change (IPCC) has employed in their recent reports on climate scientists' assessments of climate change. In recent assessments the IPCC has used verbal descriptions of uncertainty (e.g., “Likely”) accompanied by a numerical translation (e.g., in the case of “Likely,” an interval from 0.66 to 1). Budescu et al. (2014) presented participants with eight sentences from the IPCC fourth report, each containing a single probability expression. The expressions included “Likely,” “Very Likely,” “Unlikely,” and “Very Unlikely;” each of which was presented in two of the sentences. Participants were then asked to provide “lowest,” “highest,” and “best” numerical estimates of the probability they believed was intended by the authors of each sentence.

Budescu et al. (2014) found that laypeople interpret IPCC probability expressions as probabilities closer to 50% than intended by the IPCC authors. They demonstrated that an alternative presentation format that embedded the appropriate numerical range in the sentence along with the probability expression increased the correspondence between the public's interpretations and the IPCC guidelines, but still did not eradicate the regressive tendency in laypeople's interpretations.

Our interest here is in the relationship between the “best” estimates and their lower and upper counterparts, and how well the midpoint, linear-vacuous, and capital-gains models describe that relationship. The original data-set contains 10,792 responses. After eliminating those with indications that the participant may not have understood the task (those whose estimates violated the ordering lower ≤ best ≤ upper), 8665 responses were retained for the following analyses.

The three models' predictions are very strongly inter-correlated (0.963 for midpoint and linear-vacuous, 0.995 for midpoint and capital-gains, and 0.939 for linear-vacuous and capital-gains), so gross measures such as correlations are not sufficient to detect whether one model is better than another. Instead, we shall examine the root-mean-square (RMS) error between each model's predictions and the best estimates, and the model's “hit-rate,” defined as how often each model's predictions differ less than 0.05 from the best estimates (the results turn out to be insensitive to the choice of 0.05 or other nearby thresholds).

The midpoint model has the lowest RMS error, 0.081, followed by the capital-gains model with 0.093 and the linear-vacuous model with 0.123. The models' hit-rates for departing less than 0.05 from the best estimates echoes this ordering, as shown in Table 1. The midpoint model's hit-rate is 75.1%, while the capital-gains model hit-rate is 59.8% and the linear-vacuous model hit-rate is 49.1%. The format in which the probability expressions were presented has no effect on these results, and nor does the probability expression. Moreover, as is the case with the results reported by Budescu et al. (2014), these results are quite stable across countries. Both the RMS error order and hit-rate order found here hold for each of the 25 samples.

**Table 1:** **Three models' hit-frequencies**.

**CAPITAL-GAINS**				
**Midpoint**	**>0.05**	**<0.05**		
>0.05	14,084	3544	17,628	
**<0.05**	**14,431**	**38,859**	**53,290**	**Capital-gains hit-rate**
	28,515	42,403	70,918	59.8%
	Midpoint hit-rate		75.1%	
**LINEAR-VACUOUS**				
**Midpoint**	**>0.05**	**<0.05**		
>0.05	13,367	4261	17,628	
**<0.05**	**22,712**	**30,578**	**53,290**	**Linear-vacuous hit-rate**
	36,079	34,839	70,918	49.1%
	Midpoint hit-rate		75.1%	
**LINEAR-VACUOUS**				
**Capital-G**.	**>0.05**	**<0.05**		
>0.05	19,308	9207	28,515	
**<0.05**	**16,771**	**25,632**	**42,403**	**Linear-vacuous hit-rate**
	36,079	34,839	70,918	49.1%
	Capital-gains hit-rate		59.8%	

Table 1 also cross-tabulates the three pairs of models, showing that large percentages of the hits for the capital-gains and linear-vacuous models also are hits for the midpoint model, whereas the converse does not hold nearly as strongly. In the midpoint vs. capital-gains table, only 3544 out of 42,083 hits for the capital-gains model (8.4%) are not also hits for the midpoint model, whereas 14,431 out of 53,290 (27.1%) midpoint hits are not also capital-gains hits. Likewise, in the midpoint vs. linear-vacuous table, only 12.2% of the linear-vacuous hits are not also midpoint hits, whereas 42.6% of the midpoint hits are not also linear-vacuous hits. Again, the format in which the probability expressions were presented and the country or language have no effect on these patterns.

In the Budescu et al. project, participants also were asked to provide “lowest,” “highest,” and “best” numerical estimates of the probability they associate with their own interpretations of probability expressions such as “Likely,” outside of any particular context. The models' performances in predicting the context-free best estimates are very similar to what we have just seen. Again, the midpoint model has the lowest RMS error, 0.083, followed by the capital-gains model with 0.093 and the linear-vacuous model with 0.129. The midpoint model's hit-rate is 80.6%, while the capital-gains model hit-rate is 63.0% and the linear-vacuous model hit-rate is 51.0%. Finally, only 4.9% of the capital-gains model hits are not also midpoint hits, whereas 25.7% of the midpoint hits are not also capital-gains hits; and only 7.1% of the linear-vacuous hits are not also midpoint hits, whereas 41.3% of the midpoint hits are not also linear-vacuous hits. Overall, the evidence is fairly strong that the midpoint model best describes the relationship between the best estimates and their lower and upper counterparts.

### Study 2: assessing conflict and ambiguity

The study reported by Smithson (2013) examined how people make comparisons between pairs of interval estimates that are potentially both ambiguous and conflicting. This subsection summarizes that study; readers wishing for more detail may consult the 2013 paper. The study focused on four questions:

1. Do nested intervals (special case: identical midpoints) imply no conflict?
2. Do identical envelopes of intervals (i.e., the lowest and highest of their endpoints) imply equal conflict and/or equal ambiguity? What about identical interval endpoint averages?
3. Does conflict covary with the magnitudes of the differences between corresponding pairs of interval endpoints?
4. Do judgments of degrees of conflict and ambiguity both contribute independently to judgments of overall uncertainty?

The rationale for the first three questions was based on disagreements among different evidence-pooling rules, and the fourth question was motivated by the aforementioned evidence that people treat ambiguity and conflict as distinct kinds of uncertainty.

#### Method

Hypotheses and the models were tested via an online study, with a North American sample of 508 adults (205 women, 189 men, 1 unspecified; with mean age = 39.95, sd = 15.04), recruited through Qualtrics, of which 395 cases were found to be trustworthy. The experimental design had two conditions, but these proved irrelevant to the results presented here (see Smithson, 2013 for details).

**Figure 1:**
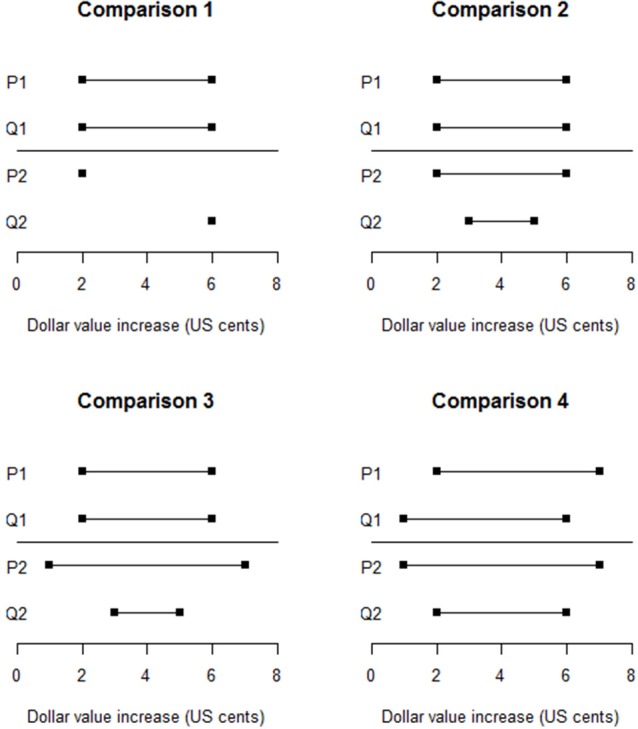
**Four pairs of judged probability intervals**.

Four comparisons between two pairs of estimates, {*P*_1_, *Q*_1_} and {*P*_2_, *Q*_2_}, were used to test questions 1–3, their results also lending insight into question 4. These comparisons are graphed in Figure 1. Comparison 1 simply tested whether people would perceive the agreeing but ambiguous intervals as more ambiguous and less conflictive than the precise but disagreeing point-estimates. Comparisons 2 and 3 tested question 1, Comparisons 3 and 4 tested question 2 (Comparison 3 because the interval endpoint averages are identical, and Comparison 4 because their envelopes are identical), and Comparisons 2–4 partially tested question 3. Participants were presented with both the graphs and verbal statements of the estimate pairs. They were asked to choose which pair of estimates exhibited more agreement, which exhibited more ambiguity, and which made them feel more uncertain about the quantity being estimated.

#### Results

As expected, in Comparison 1 a large majority of participants (84.3%) rated the two pointwise estimates as more conflictive than the identical intervals (95% CI = 80.4, 87.6%). Regarding question 1, as expected, in Comparisons 2 and 3 large majorities of respondents chose the nested interval pair as being more conflictive than the identical interval pair. An unexpected finding was that in Comparison 4, 61.5% chose the nested interval pair as more conflictive than the non-nested, overlapping pair (95% CI = 56.6, 66.2%). The results suggest that nested interval estimates are perceived as conflictive even when they have identical midpoints.

The finding regarding conflict in Comparison 4 also addresses questions 2 and 3. Thus, neither identical envelopes nor equal differences between pairs of endpoints ensures that pairs of estimates are regarded as equally conflicting. The finding for Comparison 3 demonstrates that identical average interval widths for pairs of estimates also do not ensure that they are perceived as equally conflicting.

A mixed logistic regression model utilizing the data from all four comparisons was used to address question 4, the impact of ambiguity and conflict on judgments of overall uncertainty. The model included main-effects terms for comparisons, ambiguity and agreement (conflict), with random effects for the latter two covariates. A model with interaction terms did not improve fit significantly [*X*^2^(6) = 9.642, *p* = 0.141]. Both the agreement and ambiguity terms were significant in the expected directions (a negative impact on uncertainty for agreement, *z* = −6.576, *p* < 0.0005; and positive impact on uncertainty for conflict, *z* = 12.568, *p* < 0.0005). That said, Smithson (2013) also found that judgments of ambiguity and agreement were strongly negatively related for all four comparisons (i.e., ambiguity and conflict are positively associated). The odds of choosing {*P*_1_, *Q*_1_} as the more ambiguous pair were higher if the respondent also chose {*P*_2_, *Q*_2_} as the more agreeable (and vice versa). The odds-ratios for Comparisons 1, 2, 3, and 4 were convincing: 2.90, 6.79, 14.13, and 22.84, respectively.

Finally, we turn to the performance of the models of ambiguity and conflict assessment described in the preceding section. Smithson (2013) evaluated their performance in two ways: Assigning each a “pass” or “fail” for every prediction made by each model regarding comparative conflict, ambiguity, or uncertainty; and using the differences between the scores each model assigns to every relevant pair of estimates to predict respondent choices via mixed logistic regressions. Both methods consistently indicated that, for conflict, the GV and VC2 models performed similarly and better than the VC1 model. The results for ambiguity were equivocal, with no model consistently outperforming the others. None of the models predicted the unexpected finding in comparison 4.

#### Discussion

The main findings of this study may be summarized as follows.

1. Do nested intervals (special case: identical midpoints) imply no conflict? Generally, no. In fact, in Comparison 4 people tended to judge a pair of nested intervals as more conflictive than a non-nested pair.
2. Do identical envelopes of intervals imply equal conflict and/or equal ambiguity? What about identical interval endpoint averages? No, in both cases, regarding conflict. However, the results for ambiguity were inconclusive.
3. Does conflict covary with the magnitudes of the differences between corresponding pairs of interval endpoints? No, and again the Comparison 4 result was strongly counter-indicative.
4. Do judgments of degrees of conflict and ambiguity both contribute independently to judgments of overall uncertainty? Yes, although the two-alternative forced choices for these two properties appear to be fairly strongly related. This is not necessarily an irrational association, given that there are situations where ambiguity can generate conflict or conflict can generate ambiguity.

Finally, the performance of the models suggests that the VC1 type of model is inadequate and may be abandoned. The model results also suggest that VC2 and GV need modification in at least two respects. First, Smithson (2013) reports that in Comparisons 2–4 the majority choice of which pair is more ambiguous switches depending on which pair is seen as showing more agreement. Thus, an appropriate next step would be to build and test models of conflict and ambiguity assessment that take this relationship into account.

The second recommended modification stems from the Comparison 4 finding. One interpretation of the respondents' conflict choices in Comparisons 2–4 is that some people may perceive differences in interval widths as indicating disagreement. Thus, the second pair of estimates in Comparison 4 is doubly penalized for conflict because the endpoints differ and so do the interval widths, whereas in the first pair the endpoints differ by the same amounts but the interval widths agree (i.e., the experts are equally vague). The ensuing recommendation is to amend the conflict models to accommodate a penalty for differing vagueness. Smithson (2013) made this modification and demonstrated that the augmented models' performance in the logistic regressions was substantially improved.

## Ambiguity and conflict in experienced-information settings

### Ambiguity from experience

The extension of experiments using an experienced-information setup to studies of ambiguity is quite recent, with three publications thus far (Dutt et al., 2013; Ert and Trautmann, 2014; Guney and Newell, 2014). As yet, there are no studies investigating conflictive uncertainty in the experienced-information setting. These three papers present evidence apparently showing that ambiguity aversion is reduced by sampling experience, in comparison with the levels usually found in the described-information setting.

However, each of these papers presents a different sampling method for experiencing “ambiguity.” I will argue here that none of them actually provides direct experience of ambiguity, and that this explains the decreased levels of ambiguity aversion. Ert and Trautmann's setup involves a fixed probability of a favorable outcome, and Dutt et al. (2013) has an underlying uniform distribution on the unit interval that determines the probability of a favorable outcome. These probabilities are unknown to the participants, but they learn them if they draw sufficiently large samples. Finally, the Guney–Newell (Guney and Newell, 2014) setup uses three second-order distributions for determining the probability of a favorable outcome on each turn. However, in all of these papers, the samples that participants draw from the “ambiguous” alternative are unambiguous outcomes, just like those that they would draw from an alternative whose probability has been revealed to them. The only sense in which participants could be said to be experiencing “ambiguity” is the greater variability of the outcome probabilities in the Dutt et al. and Guney–Newell setups than would be obtained by sampling events whose probabilities are fixed.

What is required instead is that participants experience ambiguous *outcomes*, i.e., the participant is left unsure about whether they have received a favorable outcome or not. Situations like this are readily found, as in inconclusive diagnostic medical examinations, “fog-of-war” occurrences in warfare, or failures to reject the null hypothesis in so-called Neyman–Pearson statistical inference.

What difference will ambiguous outcomes make to participants' judgments? Will they simply ignore them and base their judgments and decisions on the unambiguous outcomes alone? A straightforward experiment to test this would compare judgments from participants exposed to both the ambiguous and unambiguous outcomes with judgments from participants exposed to only the unambiguous outcomes.

It seems likely that participants will estimate probabilities differently when sampling ambiguous outcomes than they do when they are presented with described probability intervals. For instance, suppose we observe 45 favorable outcomes out of 60 trials, but we also have 40 trials where the outcome is not clear. What is a reasonable “best” estimate of the probability of a favorable outcome? A DM using a described-information standpoint would derive an interval of [0.45, 0.85] for this probability and then take its midpoint, 0.65, as the best estimate. However, a frequentist DM would observe that 45 out of 60 trials have been favorable where the outcomes are known, so this DM's best estimate would be the maximum likelihood estimate, 45/60 = 0.75. A Bayesian DM may arrive at yet another estimate, depending on that DM's prior.

Ironically, the frequentist DM's best estimate actually is independent of the number of trials, *N*, say. Suppose the frequentist's interval is [*f*_1_ / *N*, *f*_2_ / *N*] = [*p*_1_, *p*_2_], where *f*_1_ is the number of favorable events and *f*_2_ − *f*_1_ is the number of ambiguous events. Then the frequentist best estimate is based entirely on the unambiguous data, i.e., *f*_1_ favorable and *N* – *f*_2_ unfavorable events. The frequentist's best estimate of the probability of a favorable event is then just the ratio of favorable events to the total number of unambiguous events:

(8) $$P_{f}=\frac{f_{1}}{N-f_{2}+f_{1}}=\frac{f_{1}/N}{1-f_{2}/N+f_{1}/N}=\frac{p_{1}}{1-p_{2}+p_{1}}.$$

Comparing this result with Equation (1), we see that *P_f_* is the best estimate in the linear-vacuous mixture model.

While we have seen evidence earlier in this paper that humans are unlikely to behave as linear-vacuous (frequentist) DMs in the described-information setting, it is a reasonable hypothesis that they may do so in the experienced-information setting. It should be straightforward to design a study to compare DMs' best estimates in equivalent described- and experienced-information settings.

### Conflict from experience

Thus far, no researchers have attempted to extend the study of uncertainty in experienced-information settings to include conflicting information. There are two levels at which conflict could be experienced: diagnosis of individual trial outcomes within the same sample, and multiple samples. We will restrict attention to the individual-trial level. As I have recommended regarding ambiguity, the requirement here is that at least some of the trials be conflictive in the sense that two or more equally credible sources present opposing messages about the trial outcomes. As is the case with ambiguity, examples of conflict from experience are not difficult to find. Disagreements between medical doctors regarding a patient's diagnosis, between teachers regarding the mark for a student's essay, and among alternative sources of intelligence regarding terrorism risks are typical examples.

The psychologically relevant distinction between conflicting outcome messages and ambiguous outcomes in the setup presented here is that the DM is presented with two or more distinct sets of outcomes when there is conflict, whereas under ambiguity there is a range of possible outcomes. Returning to an earlier example, if 20 out of 50 trials have produced favorable outcomes but there are an additional 50 trials whose outcomes are unknown, the resulting interval is [0.2, 0.7]. Now suppose instead that we have two diagnoses for each trial, with agreement on 50 of them whereby 20 are favorable according to both diagnoses. If the remaining 50 trials are conflictive, such that one diagnosis says all of them are favorable whereas another says that none of them are, then we have two point-wise probability estimates, {0.2, 0.7}.

Next, suppose that of the remaining 50 trials, 20 are conflictive such that one diagnosis identifies five favorable outcomes and the second diagnosis identifies the other 15 as favorable, and the other remaining 30 trials are ambiguous. Then according to the first diagnosis the interval is [0.25, 0.55] and according to the second it is [0.35, 0.65]. In this type of setup, where the conflicting messages are restricted to “favorable” vs. “unfavorable,” the interval widths always will be identical. Differing interval widths will occur only if at least some of the conflicting messages include “ambiguous” as one of the alternatives.

The general setup is displayed in Table 2. There are two diagnoses, D_1_ and D_2_, each of which has three possible outcomes: favorable, ambiguous, or unfavorable. The cells in the table indicate the joint diagnostic outcomes (e.g., *ff* indicates that both D_1_ and D_2_ rated the outcome as favorable, and *fa* indicates that D_1_ rated the outcome as favorable and D_2_ as ambiguous). The intervals for D_1_ and D_2_ are [*f*_1_ / *N*, (*f*_1_ + *a*_1_) / *N*] and [*f*_2_ / *N*, (*f*_2_ + *a*_2_) / *N*], respectively. The special case described in the previous paragraph has *a*_1_ = *a*_2_ = *aa*, so both intervals must have identical widths. The general setup relaxes that restriction.

**Table 2:** **General conflict-ambiguity from experience setup**.

	**D_2_**				
		**Favorable**	**Ambiguous**	**Unfavorable**	
D_1_	Favorable	*ff*	*fa*	*fu*	*f*_1_
	Ambiguous	*af*	*aa*	*au*	*a*_1_
	Unfavorable	*uf*	*ua*	*uu*	*u*_1_
		*f*_2_	*a*_2_	*u*_2_	*N*

The frequentist DM constructs best estimates from these intervals in the same way as described in Equation (8). For the D_1_ and D_2_ intervals, let [*f*_1_ / *N*, (*f*_1_ + *a*_1_) / *N*] = [*p*_11_, *p*_21_] and [*f*_2_ / *N*, (*f*_2_ + *a*_2_) / *N*] = [*p*_12_, *p*_22_], respectively. The frequentist best estimate for each interval results from substituting the appropriate *p_ij_* into Equation (8).

To obtain an overall “best” estimate, a frequentist DM simply counts the number of favorable and unfavorable diagnoses by D_1_ and D_2_. The counts are shown in Table 3. The favorable-favorable cell, for instance, receives a count of 2 in the left-hand sub-table because both D_1_ and D_2_ rated those outcomes as favorable. The frequentist best estimate, given the information provided by D_1_ and D_2_, is

(9) $$P_{f}=\frac{f_{1}+f_{2}}{2N-a_{1}-a_{2}}=\frac{\left(p_{11}+p_{12}\right)/2}{1-\left(p_{21}+p_{22}\right)/2+\left(p_{11}+p_{12}\right)2}.$$

**Table 3:** **Favorable and unfavorable counts in the experienced-information setup**.

	**Favorable**				**Unfavorable**		
	**Fav**.	**Ambig**.	**Unfav**.		**Fav**.	**Ambig**.	**Unfav**.
Favorable	2	1	1	*f*_1_	0	0	1
Ambiguous	1	0	0	*a*_1_	0	0	1
Unfavorable	1	0	0	*u*_1_	1	1	2
	*f*_2_	*a*_2_	*u*_2_		*f*_2_	*a*_2_	*u*_2_

The denominator in the left-hand expression in Equation (9) is the sum of the unambiguous diagnoses returned by D_1_ and D_2_. Note that this is not the only plausible model for an overall best estimate. For instance, a DM could behave as a frequentist for best estimates in each interval and then take the midpoint between them to arrive at an overall best estimate. This setup readily generalizes to *K* diagnoses, each of which yields an interval[*p*_*k*1_, *p*_*k*2_]:

(10) $$P_{f}=\frac{\sum_{k=1}^{K}f_{k}}{KN-\sum_{k=1}^{K}a_{k}}=\frac{\sum_{k=1}^{K}p_{1k}/K}{1-\sum_{k=1}^{K}p_{2k}/K+\sum_{k=1}^{K}p_{1k}/K}.$$

In the special case where *a*_*k*_ = *aa*, *P_f_* reduces to the mean of the frequentist best estimates for each of the D_k_ intervals.

As with the frequentist best estimates of single intervals, *P_f_* in Equation (10) is a probability for which the means of the interval limits, $\sum_{k=1}^{K}p_{1k}/K$ and $\sum_{k=1}^{K}p_{2k}/K,$ are the corresponding lower and upper probabilities of Walley (1991, p. 93–94) linear-vacuous mixture. Denoting the mean of the interval widths by $\sum_{k=1}^{K}p_{2k}/K-\sum_{k=1}^{K}p_{1k}/K=\bar{w},$ from Equation (10) we have $\sum_{k=1}^{K}p_{1k}/K=\left(1-\bar{w}\right)P_{f}$ and $\sum_{k=1}^{K}p_{2k}/K=\left(1-\bar{w}\right)P_{f}+\bar{w}.$

Finally, we may consider a frequentist approach to assessing conflict and ambiguity, for comparison with the models developed in the second section of this paper. Table 4 displays the counts for ambiguity and conflict in the experienced-information setup.

**Table 4:** **Ambiguity and conflict counts in the experienced-information setup**.

	**Ambiguity**				**Conflict**		
	**Fav**.	**Ambig**.	**Unfav**.		**Fav**.	**Ambig**.	**Unfav**.
Favorable	0	1	0	*f*_1_	0	1	1
Ambiguous	1	2	1	*a*_1_	1	0	1
Unfavorable	0	1	0	*u*_1_	1	1	0
	*f*_2_	*a*_2_	*u*_2_		*f*_2_	*a*_2_	*u*_2_

The relative frequency of ambiguous diagnoses is simply

(11) $$P\left(a\right)=\left(a_{1}+a_{2}\right)/2N,$$

and the relative frequency of conflicting diagnoses is

(12) P(c)=(N−ff−aa−uu)/N.

It can be shown that *P*(*a*) is the average of the interval widths and therefore corresponds to one of the distance models in Smithson (2013), so Equation (11) does not provide a model of ambiguity assessment that differs from those in the described-information framework. However, *P*(*c*) does not correspond to any of the conflict assessment models developed by Smithson (2013), and therefore is unique to the experienced-information setting.

## Future directions

The material presented in this paper paves the way for extending our understanding of how judgments and decisions are jointly affected by conflict and ambiguity in both described- and experienced-information settings. The models of “best” estimates and subjective assessments of ambiguity and conflict can be tested and compared in both settings. They also can be utilized to predict choice behavior under conflict and ambiguity. I will conclude this paper with two additional suggestions regarding research in this domain: investigating the effects of positive vs. negative wording, and studying evaluations of and preferences for alternative ambiguous and/or conflicting estimates.

The literature on probability judgements has included investigations into the effects of wording verbal probability expressions (PEs) positively (e.g., “likely”) vs. negatively (e.g., “unlikely”). However, the analogous topic of positive vs. negative wording regarding ambiguity (e.g., “clear” vs. “unclear”) or conflict (e.g., “strongly agreeing” vs. “strongly disagreeing”) has not been studied at all. A reasonable question, then, is whether negative and positive wording could yield different effects on perceptions of ambiguity and conflict.

Several studies have found evidence that “positive” and “negative” PEs induce different actions and interpretations. Teigen and Brun (2003) show that most PEs are unidirectional and that the set of positive PEs is larger, and covers more of the probability scale, than the set of negative PEs. Teigen and Brun (2003) also find that, everything else being equal, the choice of positive or negative PEs can influence perceptions of correctness of the forecasts, surprise upon learning the outcome of events, and decisions based on these communications.

Additional effects of positive vs. negative wording were identified in Smithson et al.'s (2012) reanalysis of the Budescu's et al. (2009) data on public interpretations of PEs in the fourth IPCC report. Smithson et al.'s reanalyses revealed several findings not articulated in the Budescu et al. paper. Chief among these were considerably greater regression toward the middle of the [0, 1] interval, less consensus and poorer accuracy in translations of negative PEs than in translations of positive ones. Consequently, the variability was greater in the negatively-worded PEs (1) because the mean response was more regressive, and (2) because of greater response variation that could not be accounted for by the difference between means alone. Both of these findings suggest that people find negatively-worded PEs more ambiguous than positively-worded PEs, and that there is greater disagreement among them about the meanings of negatively-worded PEs. These results regarding positive vs. negative PEs can be applied to investigations of positive vs. negative wording of phrases referring to ambiguity and conflict.

Turning now to evaluations and preferences, Yaniv and Foster (1995, 1997) suggested that judgments and evaluations of interval estimates are the product of two competing objectives: accuracy and informativeness. They presented evidence that people tend to prefer narrow but inaccurate interval estimates over wide but accurate ones, i.e., they value informativeness more than accuracy.

Smithson's (2013) paradigm in which participants are asked to compare two pairs of estimates (which may be both ambiguous and conflicting) could be adapted easily to study the joint effects of ambiguity and conflict on the accuracy-informativeness tradeoff. Participants would be asked to choose which pair of estimates they prefer when the correct answer is revealed to them, and to assess each pair's informativeness and accuracy. A corresponding setup in an experienced-information setting would have participants sample from an environment after they have been given alternative predictive estimates of outcome probabilities for that environment.

### Conflict of interest statement

The author declares that the research was conducted in the absence of any commercial or financial relationships that could be construed as a potential conflict of interest.
